# Nasilenie lęku i Objawów Zespołu Stresu Pourazowego u Matek a Rozwój Poznawczy Ich Przedwcześnie Urodzonych Dzieci

**DOI:** 10.34763/devperiodmed.20172104.393401

**Published:** 2018-01-02

**Authors:** Tamara Zofia Walczak, Magdalena Chrzan-Dętkoś

**Affiliations:** 1 Instytut Psychologii, Uniwersytet Gdański, Gdańsk, Polska

**Keywords:** przedwczesny poród, funkcje, wykonawcze, PTSD, premature birth, executive function, Stress Disorders, Post-Traumatic

## Abstract

**Cel:**

Poszukiwanie związku pomiędzy nasileniem lęku i objawów zespołu stresu pourazowego (PTSD) u matek a zimnymi i gorącymi funkcjami wykonawczymi ich urodzonych przedwcześnie dzieci.

**Materiał i metody:**

Badaniami objęto 20 matek oraz ich dzieci urodzone przed 32. tygodniem ciąży. Nasilenie lęku u matek zmierzono za pomocą Inwentarza stanu i cechy lęku (STAI), natomiast nasilenie objawów PTSD za pomocą Zrewidowanej skali wpływu zdarzeń (IES-R). Do pomiaru gorących i zimnych funkcji wykonawczych zastosowano zadania: Pencil tap, Balance beam, Toy sort, Toy wrap i Toy wait, które pochodzą z baterii Preschool Self-Regulation Assessment (PSRA).

**Wyniki:**

Przeprowadzone badania pokazały, że prawie połowa badanych kobiet cierpi na, co najmniej umiarkowane, nasilenie objawów PTSD. Najsilniejsze objawy związane są z czynnikiem Intruzja. Wykazano również, że niższe wyniki dzieci w zakresie gorących funkcji wykonawczych są związane z wyższym lękiem, rozumianym jako cecha u matki, oraz z nasiloną Intruzją i Unikaniem oraz ogólnym wskaźnikiem PTSD.

**Wnioski:**

1. Wśród matek prawidłowo rozwijających się wcześniaków w wieku przedszkolnym aż 45% doświadczało nasilenia objawów PTSD w stopniu co najmniej umiarkowanym. 2. Nasilenie PTSD oraz lęku związane jest u matek z gorszym rozwojem gorących funkcji wykonawczych, związanych z umiejętnością odraczania gratyfikacji i hamowania zachowań u ich dzieci. 3. Przeprowadzone badanie zwraca uwagę na konieczność monitorowania stanu psychicznego rodziców wcześniaków.

## Abstract (pl)

**Aim:**

The aim of the study was to examine the relationship between anxiety, the severity of post-traumatic stress disorder (PTSD) symptoms in mothers and hot and cool executive functions of their children who were born very prematurely.

**Material and methods:**

The group consisted of 20 mothers and their children born before 32 weeks of pregnancy. The intensity of anxiety in mothers was measured using the State-Trait Anxiety Inventory (STAI), while the severity of symptoms of PTSD was assessed using the Impact of Event Scale − Revised (IES-R). Hot and cool executive functions were measured with the following tasks: Pencil tap, Balance beam, Toy sort, Toy wrap and Toy wait, from the: Preschool Self-Regulation Assessment (PSRA) battery.

**Results:**

The study showed that almost half of the women surveyed suffer from symptoms of PTSD, at least in moderate intensity. The most intense symptoms refer to the Intrusion factor. Analysis of the results showed that lower scores in hot executive functions are associated with higher anxiety - understood as a feature of mothers, severe Intrusion and Avoidance, and also the general indicator of PTSD.

**Conclusions:**

1. Among mothers, whose prematurely born children were developing property in preschool period, as many as 45% experienced at least moderate symptoms of PTSD. 2. The intensity of PTSD symptoms and anxiety in mothers is associated with worse development of hot aspect of executive functions, related to the ability to delay gratification of their children. 3. The study draws attention to the need to monitor the mental state of a parent of prematurely born child.

## Wstęp

Wcześniactwo jest największym czynnikiem ryzyka śmierci okołoporodowej oraz poważnych uszkodzeń neurologicznych. Liczne badania [1, 2, 3] wskazują na to, że dzieci urodzone przedwcześnie, częściej niż ich rówieśnicy urodzeni o czasie, przejawiają trudności w funkcjonowaniu poznawczym. Wcześniejszy termin urodzenia, wczesne uszkodzenia rozwijającego się mózgu, są czynnikami ryzyka dla późniejszych trudności poznawczych [2]. Z drugiej strony, badania wskazują również na znaczenie jakości wczesnych interakcji w diadzie rodzic-dziecko [1, 2].

Matki wcześniaków doświadczają znaczniej częściej objawów zespołu stresu pourazowego (PTSD) i stresu rodzicielskiego. W przypadku porodów terminowych, PTSD stwierdza się u mniej niż 8 % rodzących w ciągu 6 tygodni po porodzie i odsetek ten zmniejsza się w czasie [4]. W badaniu zespołu Feeley [5], PTSD doświadczało ponad 23% matek niemowląt urodzonych przedwcześnie. Praca zespołu Goutaudiera [6] wykazała, że według *Zrewidowanej skali wpływu zdarzeń* (IES-R) PTSD doświadcza aż 77% matek wcześniaków. W badaniu zespołu Shaw [7] 1/3 matek otrzymała diagnozę PTSD miesiąc po wypisie ze szpitala. Co więcej, wskazuje się [8], że poziom stresu pourazowego u matek wcześniaków nie maleje wraz z upływem czasu. Ponadto, percepcja przedwczesnego porodu jako wydarzenia traumatycznego może prowadzić do rozwinięcia się stresu rodzicielskiego.

Prace zespołu Pierrehumbert [9] wskazują na związek występowania objawów PTSD u matek, z trudnościami w samoregulacji u małych dzieci: wzorcami jedzenia i spania. Trudności te tylko w części tłumaczone mogą być przez czynniki okołoporodowe – istotny predyktor stanowią nasilone objawy PTSD. Nadopiekuńczość oraz wysoki poziom lęku u matek wcześniaków może utrudniać tworzenie atmosfery sprzyjającej optymalnemu rozwojowi dzieci. Barratt, Roach, i Leavitt [10] zaobserwowali, że mimo podobnego rozwoju poznawczego i emocjonalnego dzieci w obu grupach, matki zdrowych wcześniaków były w stosunku do nich bardziej kontrolujące.

Badania Brzezińskiej i Nowotnik [11] pokazują, że stosunek emocjonalny do dziecka i styl sprawowania rodzicielskiej kontroli są czynnikami wpływającymi na rozwój funkcji wykonawczych (ang. *executive function*, EF) dziecka: kompetencji związanych m.in. z pamięcią operacyjną, uwagą, hamowaniem oraz z elastycznością poznawczą. EF umożliwiają samoregulowanie własnego zachowania. Wyróżnia się tzw. *gorące* EF – oceniające zdolność regulacji emocji oraz wzorce motywacyjne dziecka oraz *zimne* EF – związane z rozwiązywaniem problemów opartych o materiał abstrakcyjny, taki jak pojęcia czy symbole [12, 13]. Większe trudności w zakresie kompetencji wykonawczych u wcześniaków obserwuje się w grupie dzieci urodzonych skrajnie wcześnie [14]. Mimo prawidłowego rozwoju jedną z najczęstszych trudności w grupie wcześniaków, są problemy z koncentracją uwagi [15, 16, 17]. Badania oceniające funkcjonowanie dzieci, urodzonych przed 32. tygodniem ciąży (t.c.), w dzieciństwie, adolescencji i dorosłości, ukazują występujący u nich trwały wzorzec zachowań i trudności w obszarze: selektywnej uwagi, zmiany uwagi, jak i wytężonej uwagi [15, 14]. Johnson i Marlow [18] opisali wzorzec zachowania wcześniaków, który określili jako „fenotypowy”. Charakteryzuje się on zwiększonym ryzykiem trudności w koncentracji uwagi, lękiem i problemami w funkcjonowaniu społecznym [18]. Badania Salonen, Leopla i Vauras [19] oraz zespołu Lucassen [20] wykazały, że oddziaływania wychowawcze istotnie związane są z funkcjami wykonawczymi dziecka – m.in. mniejsza wrażliwość matek związana jest gorszym hamowaniem zachowania u dzieci [19].

## Cel pracy

Celem badania była analiza związku pomiędzy lękiem i nasileniem objawów PTSD u matek, a funkcjami wykonawczymi ich przedwcześnie urodzonych dzieci. Podjęto próbę odpowiedzi na pytanie o to, czy gorsze funkcjonowanie w zakresie *gorących* i *zimnych* funkcji wykonawczych u wcześniaków, jest związane z większym nasileniem lęku i objawów PTSD u matek.

## Materiał i metody

Przy planowaniu badania założono następujący model teoretyczny, wyjaśniający poszczególne zależności między zmiennymi (ryc. 1).

### Osoby badane i procedura

Grupę badaną stanowiły matki (N=20) w wieku od 27 do 46 lat, których średni wiek wynosił 36 lat (*SD*=4,47). Większość matek zadeklarowała posiadanie wykształcenia wyższego (90,00%). W badaniu wzięły również udział ich dzieci (N=20), które urodziły się przed 32. tygodniem ciąży. Średni wiek ciążowy wynosił 29,50 tygodni, mediana – 30,00, minimum – 26 i maksimum – 31 tygodni. Rozrzut masy urodzeniowej wynosił: minimum – 600, maksimum – 1740 gramów, ze średnią – 1169,22 i medianą 1167,50 gramów. Dane pochodzące z wypisów ze szpitala, książeczek zdrowia dzieci i wywiadu z rodzicami, zawierały informacje o wylewach do OUN u 7 badanych dzieci (n=3 – wylew 3. stopnia, n=3 – wylew 2. stopnia, n=1 – wylew 1. stopnia), retinopatii wcześniaczej u 6 dzieci, zespole zaburzeń oddychania u 9 dzieci i niedokrwistości u 11 dzieci. Ponadto, wypisy ze szpitala zawierały informacje o występujących torbielach w mózgu u 3 dzieci. Kryterium wykluczające udział w badaniu obejmowało ciężkie wady wrodzone i niepełnosprawność ruchową dziecka, która uniemożliwiała mu swobodne poruszanie. Ponadto z uwagi na rozpiętość wieku badanych dzieci, nie było możliwe zbadanie całościowego ich rozwoju jednym narzędziem. Z informacji uzyskanych w wywiadzie z rodzicami wynika, że 19 z 20 przebadanych dzieci uczęszczało do przedszkola publicznego. Również obserwacja dzieci podczas swobodnej zabawy i właściwego badania wykazała, że u dzieci w tej grupie nie obserwuje się widocznych deficytów rozwojowych.

W momencie badania wcześniaki były w wieku od 36 do 71 miesięcy, ze średnią – 51,55 miesięcy (*SD*=11,90). W grupie dominowały dziewczynki (60,00%).

Badania prowadzono na przełomie lat 2015/2016 w Instytucie Psychologii Uniwersytetu Gdańskiego, po uzyskaniu zgody komisji etyki badań naukowych. Matki zostały proszone o wypełnienie *Inwentarza stanu i cechy lęku* (STAI) oraz *Zrewidowanej skali wpływu zdarzeń* (IES-R), natomiast dzieci zaproszono do badania w sali wyposażonej w lustro weneckie. Pojedyncze badanie matki i jej dziecka trwało średnio ok. 1,5 godziny.

### Metoda

Do pomiaru funkcji wykonawczych wykorzystano, za zgodą jej twórców, skalę *Preschool Self-Regulation Assessment* (PSRA). Skala, została opracowana przez Smith-Donald, Raver, Hayes i Richardson w 2007 roku na Uniwersytecie Chicago i służy pomiarowi zdolności samoregulacyjnych dzieci w standaryzowanej sytuacji badawczej [21]. Do pomiaru *zimnych* funkcji wykonawczych wykorzystano zadania: *Pencil tap* i *Balance beam*. *Gorące* funkcje oceniono na podstawie zadań: *Toy sort*, *Toy wrap* i *Toy wait* [12]. W zadaniu *Pencil tap* wynik końcowy stanowi liczba prawidłowych stuknięć ołówkiem, w *Balance beam* jest to różnica czasu między próbami swobodnego i wolnego przejścia po prostej (im większa różnica, tym lepszy wynik). W zadaniu *Toy Sort* miarę stanowi czas wykonania zadania przez dziecko, po wydaniu polecenia (im krótszy czas, tym lepszy wynik). W zadaniach *Toy wrap* i *Toy wait* mierzony jest czas, w którym dziecko stosuje się do wydanego polecenia (im dłuższy czas, tym lepszy wynik).

**Ryc. 1:**
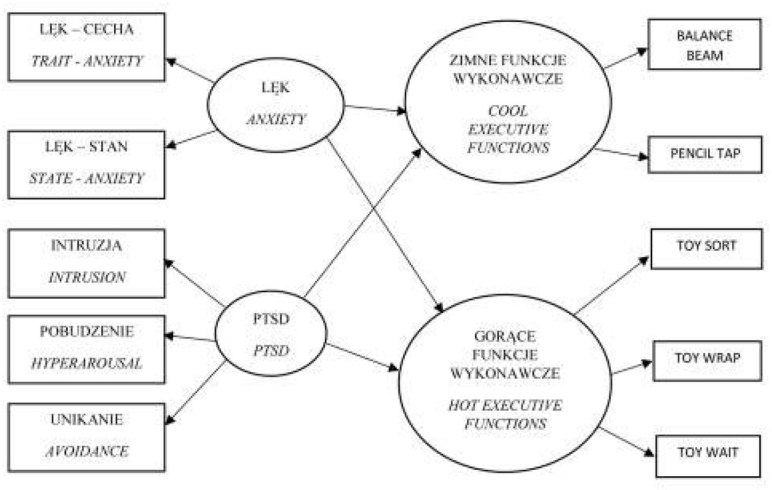
Teoretyczny model zależności testowanych w badaniu (opracowanie własne). Fig. 1. The theoretical model of dependencies tested in the study (own study).

Nasilenie lęku u matek sprawdzono *Inwentarzem stanu i cechy lęku* (STAI), autorstwa Spielbergera, Gorsucha i Lushene’a, w polskiej adaptacji Spielbergera, Strelaua, Tysarczyk i Wrześniewskiego [22]. Kwestionariusz złożony jest z 20 pytań, składających się na dwie skale: X-1, która służy do badania *lęku-stanu* oraz X-2, do badania *lęku-cechy* [22].

Do pomiaru nasilenia objawów potraumatycznych u matek wykorzystano *Zrewidowaną skalę wpływu zdarzeń* (IES-R), autorstwa Weissa i Marmara, w polskiej wersji opracowanej przez Juczyńskiego i Ogińską-Bulik [23]. Skala jest wykorzystywana do monitorowania zmian w nasileniu reakcji potraumatycznych i przeznaczona jest przede wszystkim do celów badawczych (znajduje też zastosowanie w praktyce w badaniach przesiewowych i profilaktycznych). Składa się z 22 pozycji (stwierdzeń), które tworzą trzy podskale (czynniki): *Intruzja*, *Pobudzenie* i *Unikanie*. Łączny wynik dla wszystkich podskal określany jest jako *wskaźnik PTSD*.

Obliczenia statystyczne zostały wykonane przy pomocy programu IBM SPSS for Windows na licencji Uniwersytetu Gdańskiego. Zastosowano metodę wielorakiej regresji liniowej (ang. *Multiple Linear Regression*), poprzedzoną analizą macierzy korelacji Pearsona (ang. *Pairwise Pearson Correlation*).

## Wyniki

Wyniki dotyczące matek w zakresie czynnika *lękstan* (M=5,20, SD=2,53) i czynnika *lęk-cecha* (M=5,60, SD=2,19) nie odbiegają statystycznie istotnie od średniej w populacji (tab. I). W tabeli II ukazano wyniki nasilenia PTSD w zakresie: *Intruzji* (*M=*1,70, *SD*=0,26), *Pobudzenia* (*M=*1,32, *SD*=0,25) i *Unikania* (*M=*0,89, *SD*=0,18), a także ogólnego *wskaźnika PTSD* (*M=*1,32, *SD*=0,93). Średni wynik matek w zakresie czynnika *Intruzja* znajduje się powyżej wartości granicznej (1,5) dla objawów PTSD, co świadczyć może o co najmniej *umiarkowanym* nasileniu objawów. Należy również dodać, że aż dziewięć z badanych 20 kobiet uzyskało we wskaźniku PTSD wynik powyżej punktu odcięcia. W tabeli III pokazano istotną statystycznie korelację *lęku-cechy* z zadaniem *Toy Sort* (*r*=0,53), które mierzy *gorące* funkcje wykonawcze. Jak wcześniej wspomniano, w zadaniu *Toy sort* wyższy wynik oznaczał gorsze funkcjonowanie, zatem gorsze funkcjonowanie w zakresie *gorącego* aspektu funkcji wykonawczych, jest związane z wyższym lękiem, rozumianym jako cecha u matki (tab. III).

**Tabela I:** Wyniki STAI w grupie matek. Table I. The results of STAI in the group of mothers.

Wyniki stenowe STAI *Sten results of STAI*	Statystyki opisowe *Descriptive statistics*			Test istotności różnic *Test of significance*			
	M	SD	N	t	df	p	Z
Lęk – stan/ *State anxiety*	5,20	2,53	20	-,534	19	,601	-,12
Lęk – cecha/ *Trait Anxiety*	5,20	2,19	20	-,612	19	,548	-,14

**Tabela II:** Wyniki IES-R w grupie matek. Table II. The results of IES-R in the group of mothers.

Wyniki IES-R *Results of IES-R*	M	SD	Min	Max
Intruzja/Intrusion	1,70	,26	0	3,88
Pobudzenie/Hyperarousal	1,32	,25	0	3,71
Unikanie/Avoidance	,89	,18	0	3,71
Wskaźnik PTSD/PTSD indicator	1,32	,93	0	3,18

**Tabela III:** Korelacja (r-Pearsona) wyników PSRA w grupie wcześniaków z wynikami STAI w grupie matek. Table III. The correlation (Pearson’s r) between PSRA battery among premature children with the results of STAI in the group of mothers.

Zadanie *Task*	Lęk − Stan *State anxiety*	Lęk − Cecha *Trait Anxiety*
Balance Beam	,39	,32
Pencil Tap	-,33	-,39
Toy Sort	,30	,53*
Toy Wrap	-,36	-,41
Toy Wait	,06	-,20

*Korelacja jest istotna na poziomie p < 0.05/*Statistically significant difference at p < .05*

Z tabeli IV wynika, że *Pobudzenie* oraz *wskaźnik PTSD* statystycznie istotnie korelują z wynikami zadania *Balance beam* (*r*=0,57, *r*=0,50), które mierzy *zimne* funkcje wykonawcze. Ponadto *wskaźnik PTSD*, *Intruzja* i *Unikanie* statystycznie istotnie korelują z zadaniem *Toy sort* (*r*=0,51, *r*=0,47, *r*=0,52), które mierzy *gorące* funkcje wykonawcze. Oznacza to, że wyższe nasilenie tych dwóch komponentów oraz *wskaźnika PTSD* u matek, wiąże się z gorszym funkcjonowaniem w zakresie *gorących* funkcji wykonawczych u dzieci (w zadaniu *Toy sort* wyższy wynik oznaczał gorsze funkcjonowanie). Zaskakujące może natomiast wydawać się to, że wyższe nasilenie *Pobudzenia* i ogólnego *wskaźnika PTSD* u matek wiąże się z lepszym funkcjonowaniem ich dzieci w zakresie *zimnych* funkcji wykonawczych (w zadaniu *Balance beam* wyższy wynik oznaczał lepsze funkcjonowanie).

**Tabela IV:** Korelacja (r-Pearsona) wyników PSRA w grupie wcześniaków z wynikami IES-R w grupie matek. Table IV. The correlation (Pearson’s r) between PSRA battery among premature children with IES-R in the group of mothers.

Zadanie *Task*	Intruzja *Intrusion*	Pobudzenie *Hyperarousal*	Unikanie *Avoidance*	Wskaźnik PTSD *PTSD indicator*
Balance Beam	,44	,57**	,29	,50*
Pencil Tap	-,02	-,14	-,26	-,13
Toy Sort	,51*	,41	,47*	,52**
Toy Wrap	,04	-,08	-,19	-,06
Toy Wait	,23	,28	,35	,30

*Korelacja jest istotna na poziomie p < 0.05/*Statistically significant difference at p < .05*.

**Korelacja jest istotna na poziomie p < 0.01/*Statistically significant difference at p < .01*.

Przeprowadzona analiza regresji ujawniła, że jedynie *lęk jako cecha* stanowić może istotny (*p<*0,05) predyktor funkcjonowania wykonawczego dzieci, w zakresie aspektu *gorącego*, mierzonego zadaniem *Toy Sort*. W przypadku *zimnych* funkcji wykonawczych oraz *gorących* mierzonych zadaniami *Toy Wait i Toy Wrap*, siła zależności jest słaba i statystycznie nieistotna. Szczegółową analizę modelu regresji zawarto w tabeli V.

**Tabela V:** Szczegółowa analiza modelu regresji dla lęku jako cechy i stanu oraz gorących i zimnych funkcji wykonawczych. Table V. The detailed analysis of the regression model for trait-state anxiety and hot and cool executive functions.

Zadanie/Task																				
Zimne funkcje wykonawcze/*Cool executive functions*									Gorące funkcje wykonawcze/*Hot executive functions*											
	Balance Beam				Pencil Tap				Toy Sort				Toy Wrap				Toy Wait			
	*B*	*SE*	*r^2^_p_*	*P*	*B*	*SE*	*r^2^_p_*	*P*	*B*	*SE*	*r^2^_p_*	*P*	*B*	*SE*	*r^2^_p_*	*P*	*B*	*SE*	*r^2^_p_*	*P*
Lęk - stan *State anxiety*	,136	,128	,06	,151	-,06	,18	,01	,370	-,25	,53	,01	,318	-,36	,73	,01	,313	,65	,51	,08	,109
Lęk - cecha *Trait Axiety*	,045	,166	,00	,395	-,24	,24	,05	,163	1,50	,68	,20	,021	-,92	,95	,05	,173	-1,01	,66	,12	,072
	R^2^=,396; F(2; 17)=1,584; p=,234				R^2^=,397; F(2; 17)=1,589; p=,233				R^2^=,541; F(2; 17)=3,513; p=,053				R^2^=,521; F(2; 17)=1,826; p=,191				R^2^=,354; F(2; 17)= 1,219; p=,320			

W dalszej analizie regresji podjęto próbę sprawdzenia, czy nasilenie objawów PTSD u matek stanowić może dobry predyktor funkcjonowania wykonawczego wcześniaków. Z powodu współliniowości z analizy usunięto czynnik *Pobudzenie*. Analiza regresji ujawniła, że *Intruzja i Unikanie*, stanowią istotne (*p<*0,05) predyktory funkcjonowania wykonawczego dzieci w zakresie *gorącego* aspektu, mierzonego zadaniem *Toy Sort*. W przypadku *zimnych* funkcji wykonawczych oraz *gorących* mierzonych zadaniami *Toy Wait i Toy Wrap* siła zależności jest słaba i statystycznie nieistotna. Szczegółową analizę modelu regresji odnaleźć można w tabeli VI.

**Tabela VI:** Szczegółowa analiza modelu regresji dla PTSD oraz gorących i zimnych funkcji wykonawczych. Table VI. The detailed analysis of the regression model for PTSD and hot and cool executive functions.

Zadanie/*Task*																				
Zimne funkcje wykonawcze/*Cool executive functions*									Gorące funkcje wykonawcze/*Hot executive functions*											
	Balance Beam				Pencil Tap				Toy Sort				ToyWrap				Toy Wait			
	B	SE	r^2^_p_	P	B	SE	r^2^_p_	P	B	SE	r^2^_p_	P	B	SE	r^2^_p_	P	B	SE	r^2^_p_	P
Intruzja *Intrusion*	1,53	,99	,11	,071	1,01	1,50	,02	,256	5,98	4,14	,08	,084	4,81	6,20	,03	,224	,50	4,04	,00	,452
Unikanie *Avoi-dance*	,27	1,46	,00	,427	-2,87	2,21	,09	0,106	6,53	6,08	,05	,149	-10,02	9,12	,07	,144	7,00	5,94	,07	,128
	R^2^=,196; F(2; 17)=2,077; p=,156				R^2^=,090; F(2; 17)=0,845; p=,447				R^2^=,309; F(2; 17)=3,795; p=,043				R^2^=,068; F(2; 17)=0,618; p=,550				R^2^=,122; F(2; 17)= 1,184; p=,330			

*Z analizy usunięto predyktor: *Pobudzenie*, z powodu współliniowości/*Predictor*: *Agitation was removed from the analysis, because of the collinearity problem*.

## Dyskusja

Ekstremalnie wczesny poród to nie tylko trauma biologiczna dla dziecka, ale również wydarzenie o potencjale traumatycznym dla jego rodziców. Przegląd literatury oraz wyniki badań własnych pokazują, że konsekwencje wcześniactwa nie kończą się wraz z wypisem ze szpitala. Wykazano, że u matki konsekwencje emocjonalne przedwczesnego porodu nie mijają w pierwszym roku życia, a są obserwowane nawet w okresie przedszkolnym. W badaniu zespołu Goutaudiera [6] PTSD można było zdiagnozować u 77% matek, w badaniu własnym odsetek ten wynosił 45%.

Nasilenie objawów potraumatycznych u matek dzieci urodzonych przed 32. t.c. wydaje się być związane z niższymi kompetencjami w zakresie regulacji emocjonalnej u ich dzieci. Należy przy tym podkreślić możliwą dwukierunkowość tej relacji. Z jednej strony lęk i nasilenie objawów PTSD mogą wpływać na mniejszą responsywność matki na potrzeby dziecka (od początku relacji w tej diadzie), co przekładać się może na niższą wrażliwość macierzyńską (zdolność do odnoszenia się i odpowiedniego reagowania na sygnały płynące od dziecka [11]). Umiejętność ta jest kluczowa dla rozpatrywania znaczenia stosunku emocjonalnego matki do dziecka, a także charakterystycznego dla niej stylu sprawowania matczynej kontroli. Z drugiej strony źródłem podwyższonych wyników w zakresie PTSD oraz lęku, mogą być rozwojowe skutki wcześniactwa, z którymi bardzo często muszą radzić sobie rodzice. Te dwa poruszone aspekty mogą wiązać się z trudnościami w zakresie funkcji wykonawczych dziecka, a wtórnie także z mniejszymi osiągnięciami szkolnymi i trudnościami w aspekcie adaptacyjnym i motywacyjnym [24].

O ile związek nasilenia *lęku-cechy* oraz *Intruzji* i *Unikania* z funkcjonowaniem wykonawczym potwierdza założenia teoretyczne, to związek *Pobudzenia* oraz ogólnego *wskaźnika PTSD* z tym konstruktem był po części zaskakujący. Być może *zimne* funkcje wykonawcze należałoby rozpatrywać jako niezależny konstrukt, którego poziom zależy w mniejszym stopniu od czynników o charakterze psychologicznym, a bardziej od zdolności w rozumieniu np. inteligencji płynnej. Niemniej badania w tym zakresie wymagają dalszych poszukiwań i próby wyjaśnienia tej zależności, np. przez rozważenie innych czynników takich jak wzrost potraumatyczny u matek, czy jakość środowiska rodzinnego.

Poruszony problem badawczy wydaje się istotny dla planowania oddziaływań skierowanych na pomoc rodzicom. Badania wskazują na wysoką skuteczność stosunkowo krótkich, zmanualizowanych interwencji dla rodziców wcześniaków – przykładem jest randomizowana próba kliniczna opisana przez zespół Shaw [25]. Interwencja ta bazuje na psychoedukacji, nauce relaksacji, poznawczej restrukturyzacji wydarzenia oraz możliwości zwerbalizowania doświadczanych uczuć. Dodatkowo istotnym elementem krótkoterminowej terapii jest praca nad percepcją dziecka i opracowywaniem doświadczenia bycia rodzicem wcześniaka. Rodzice, którzy brali udział w krótkoterminowej terapii doświadczyli istotnego obniżenia objawów depresji, PTSD oraz rodzicielskiego stresu. Warto zatem, aby specjaliści pracujący z wcześniakami: lekarze neonatolodzy, rehabilitanci, logopedzi, pedagodzy, psychologowie, zdawali sobie sprawę z częstotliwości nasilenia objawów pourazowych u matek wcześniaków i byli przygotowani do ich identy&kowania. W niektórych sytuacjach zajęcie się rodzicami byłoby istotnym elementem pro&laktyki prawidłowego rozwoju dziecka. Wczesna interwencja, mająca na celu wspieranie matki w tworzeniu bardziej zrównoważonego i zróżnicowanego postrzegania swojego dziecka, może zminimalizować potencjalne szkodliwe skutki dla relacji rodzic-dziecko oraz dla przyszłego rozwoju dziecka (w tym rozwoju poznawczego).

Największym ograniczeniem badania własnego jest małoliczna grupa, która uniemożliwiła włączenie do modelu np. ryzyka medycznego. Planując kolejne badania warto zwiększyć liczbę osób badanych, a najlepiej przeprowadzić badania longitudinalne, obejmujące kilka okresów rozwojowych. Umożliwiłoby to prześledzenie trajektorii rozwoju omawianych funkcji u wcześniaków. Natomiast zróżnicowanie wieku ciążowego umożliwiłoby włączenie go do modelu badawczego, jako dodatkowego czynnika.

## Wnioski

1. Wśród matek prawidłowo rozwijających się wcześniaków w wieku przedszkolnym aż 45% doświadczało nasilenia objawów PTSD w stopniu co najmniej umiarkowanym.
2. Nasilenie PTSD oraz lęku związane jest u matek z gorszym rozwojem gorących funkcji wykonawczych związanych z umiejętnością odraczania graty&kacji i hamowania zachowań u ich dzieci.
3. Przeprowadzone badanie zwraca uwagę na konieczność monitorowania stanu psychicznego rodziców wcześniaków.
